# Effects of Different Water Contents on the Quality Characteristics of Roasted Large Yellow Croaker (*Larimichthys crocea*) Fillets

**DOI:** 10.3390/foods14091638

**Published:** 2025-05-07

**Authors:** Shuting Huang, Shuji Liu, Ping Wen, Xiangyang Lin, Xiaoting Chen, Yongchang Su, Yuping Xie, Huawei Zheng, Yihui Chen, Zhiyu Liu

**Affiliations:** 1 College of Food Science, Fujian Agriculture and Forestry University, Fuzhou 350002, China; 18065690122@163.com (S.H.); harris2197395@163.com (Y.C.); 2 Fisheries Research Institute of Fujian, Xiamen 361013, Chinaxtchen@jmu.edu.cn (X.C.); suyongchang@stu.hqu.edu.cn (Y.S.); 3 Xiapu County Marine Fisheries Development Center, Ningde 355100, China; 4 College of Geography and Oceanography, Minjiang University, Fuzhou 350002, China; zhw@mju.edu.cn; 5 Key Laboratory of Cultivation and High-Value Utilization of Marine Organisms in Fujian Province, National Research and Development Center for Marine Fish Processing (Xiamen), Xiamen 350002, China

**Keywords:** large yellow croaker, water content, roasting, quality characteristics, flavor profile

## Abstract

This study investigated the effect of the water content of large yellow croaker fillets on their quality characteristics after roasting. The large yellow croaker fillets were randomly divided into groups, namely, the fresh group (BMC-77), the 3% salt-cured group (BMC-70), and groups cured with 3% salt followed by hot air drying to obtain different moisture contents (BMC-65, BMC-60, and BMC-55). Then, the fillets were roasted at 220 °C for 20 min. There were four replicates for each group. Various indicators, including color, texture, thiobarbituric acid-reactive substance (TBARS) content, total volatile basic nitrogen (TVB-N) content, water distribution, volatile components, and myofibrillar proteins were determined, and a sensory evaluation was carried out. The results showed that as the water content decreased, the lightness (L*) of the roasted fillets significantly decreased (*p* < 0.05), while the redness (a*) and yellowness (b*) increased. The hardness, shear force, TBARS, and TVB-N values all increased significantly (*p* < 0.05). The proportion of immobile water decreased, while the proportions of tightly bound water, free water, and loosely bound water increased. The electronic nose, electronic tongue, and GC-MS analyses revealed that there were significant differences in odor, taste, and volatile components among fillets with different water contents. A comprehensive analysis of all the indicators demonstrated that the fillets with an initial water content of 65% (BMC-65) achieved the best sensory qualities after roasting in terms of taste and flavor. An appropriate reduction in the initial water content helped to improve the texture and appearance of the fillets while delaying the degradation of proteins and lipids. This study provides a theoretical foundation for optimizing the roasting process of large yellow croaker fillets. Future research could explore the synergistic effects of the roasting conditions and water content to achieve more accurate quality control.

## 1. Introduction

The large yellow croaker (*Larimichthys crocea*), which belongs to the genus Corynoscidae of Perciformes, is an important marine economic fish. It is popular among consumers due to its delicate meat and delicious taste. With the continuous advancement of aquaculture technology, the output of large yellow croaker catches has increased significantly. At present, the large yellow croaker has become the marine aquaculture fish with the highest production in China. According to statistics, the output of the large yellow croaker in China reached 281,000 tons in 2023 [1].

However, at present, the processing scale for the large yellow croaker is not large, which mainly involves traditional methods such as freezing, refrigeration, curing, and drying. Freezing and refrigeration can effectively extend the shelf life of large yellow croaker fish but will affect their taste and flavor to a certain extent [2]. Adding salt, sugar, spices, and other seasonings to pickled large yellow croakers gives them a unique flavor [3]; however, if the curing process is not properly controlled, it may lead to excessive levels of salt, which can affect the health of consumers. Dried large yellow croaker can be preserved for a long time, which is convenient for transportation and sales. Nevertheless, some nutrients will be lost during the drying process, and it will become relatively tough [4].

In recent years, with the continuous increase in consumer requirements for food quality and taste, some new processing methods have gradually emerged. Among them, roasting has drawn much attention [5]. Liu et al. [6] compared the (non)volatile compounds and sensory qualities of oyster cuts of roasted lamb treated with three newer roasting methods. Duppeti et al. [7] researched the biochemical composition, color, and nonvolatile taste active compounds of roasted whiteleg shrimp. Nie et al. [8] indicated that pre-marination with tea significantly improved the flavor of roasted fish. Roasting creates a crispy golden crust on the surface of large yellow croaker fillets while maintaining a tender and juicy texture inside and imparting a distinctive smoky flavor [9]. However, different processing conditions, especially differences in water content, will have significant impacts on the quality characteristics of large yellow croaker fillets after roasting [10].

As an important physical property of fish fillets, the water content is directly related to moisture migration, heat transfer, and chemical reactions during processing [11]. During the pre-roasting process, fish fillets with different water contents will undergo different water evaporation rates, as well as protein denaturation and lipid oxidation processes, which will further affect the quality of the final product [12]. The Maillard reaction is an important nonenzymatic browning reaction in food processing, which has a key impact on the color, flavor, and nutritional content of food [13]. Moisture is a key factor influencing the Maillard reaction. The right amount of water has a promoting effect on the Maillard reaction in fish [14]. During the roasting process, a certain moisture content aids in the migration of sugars and amino acids inside the fillet, allowing the reaction to occur over a wider area, resulting in a more uniform production of Maillard pigments and flavor compounds, and giving the fillet a good color and attractive aroma [15,16]. For large yellow croaker fillets, an appropriate water content can ensure that they are evenly heated during the roasting process to obtain a good taste and flavor. Either too much or too little moisture can negatively affect the Maillard reaction [17]. Excessive moisture will dilute the concentration of reactants, reduce the effective collision frequency between reactants, and lead to excessive heat transfer, which is not conducive to the progress of the reaction and may make the fish fillets over-cooked during the roasting process, affecting the texture and taste of the fish fillets [18]. A high water content may also lead to excessive wetness on the surface of the fillets during the roasting process; if the surface of the fish fillet is too wet during the baking process, the fillets become prone to breaking up into garlic-clove-like pieces, and it becomes difficult to form a crisp crust [19,20]. On the other hand, if the water content is too low, it may cause the fillet to become overly dry during the roasting process, resulting in a hard and brittle texture, and it loses its tender and juicy characteristics [21]. However, excessive heating may also lead to a loss of the fish flavor itself. Therefore, drying and roasting are both important processing methods. By drying large yellow croaker fillets, the moisture content can be appropriately reduced, shortening the roasting time, reducing the formation of some heterocyclic amines and carcinogens during the roasting process, and promoting the Maillard reaction [22].

Some progress has been made in the research on the quality characteristics of fish fillets during the pre-roasting process. Scholars at home and abroad have conducted in-depth studies on multiple aspects such as raw material selection and processing techniques. For instance, Oluwaniyi et al. [23] used four types of fish—namely, *Clupea harengus*, *Scomber scombrus*, *Trachurus trachurus*, and *Urophycis tenuis*—as raw materials to study the effects of different methods such as frying and roasting on the amino acid content of fish meat. Yin et al. [9] investigated the impact of different roasting temperatures on the quality of fish fillets and found that although high-temperature roasting shortened the processing time, it led to a harder texture and impaired flavor of the fish fillets. Zhang et al. [24] analyzed the influence of different roasting times on the quality of fish fillets and discovered that an overly long roasting time resulted in excessive dehydration and loss of flavor of the fish fillets.

At present, most studies focus on a specific link or a certain quality indicator when studying the roasting process of fish fillets, and there is a lack of systematic analyses and comparisons of the quality characteristics of fish fillets with different water contents, with little research on the large yellow croaker in particular. Zhu et al. [25] investigated the effects of four drying methods on the meat quality of semi-dried Takifugu obscurus fillets. Jiang et al. [26] studied the effects of different heat treatments such as steaming, boiling, roasting, and microwaving on the fatty acids, volatile flavors, and flavor compounds of channel catfish. In recent years, a small number of studies have focused on the processing of large yellow croaker fillets. Li et al. [27] studied the quality changes of large yellow croaker fillets during refrigeration. Cui et al. [28] investigated the relationship between the lipid composition and characteristic flavor of steamed large yellow croaker. However, research on the effects of pre-drying and pre-roasting processes on the quality characteristics of large yellow croaker fillets is still insufficient. Therefore, it is difficult to comprehensively determine how the quality characteristics of large yellow croaker fillets change during the roasting process.

Thus, this study aimed to reduce the water content of large yellow croaker fillets through a drying pre-treatment and then systematically analyzed the changes in the quality characteristics of large yellow croaker fillets with different water contents during the roasting process to provide a theoretical basis and technical support for the development of high-quality roasted large yellow croaker fillets.

Through this study, we aimed to determine how different water contents affect the quality characteristics of large yellow croaker fillets during the roasting process and provide a scientific basis for further optimizing the processing technology to improve product quality. Meanwhile, this study provides useful references and cases for research on the quality characteristics during the roasting process of large yellow croaker slices and the quality control of other aquatic products, promoting the sustainable development of the aquatic processing industry.

## 2. Materials and Methods

### 2.1. Materials and Reagents

Fresh farmed large yellow croakers were purchased from Ningde Yihai Aquatic Products Co., Ltd., Ningde, China. A PAGE Gel Rapid Preparation Kit (12.5%) was purchased from Shanghai Yamei Biomedical Technology Co., Ltd., Shanghai, China. Trichloroacetic acid (analytical grade), anhydrous ethanol (analytical grade), sodium chloride (analytical grade), sodium hydroxide (analytical grade), 2-thiobarbituric acid, boric acid, sulfuric acid, bromocresol green, methyl red (analytical grade), and other chemicals were all purchased from Sinopharm Chemical Reagent Co., Ltd., Shanghai, China.

### 2.2. Equipment

A BGZ-240 Electric Heating Blowing Drying Oven and ADCI Series Automatic Colorimeter were purchased from Beijing Chentaike Instrument Technology Co., Ltd., Beijing, China. A TA-XTplus Texture Analyzer was purchased from Stable Micro Systems in London, UK. A MesoMR Nuclear Magnetic Resonance Analysis and Imaging System was provided by Shanghai Niumai Electronic Technology Co., Ltd., Shanghai, China. A PEN3.5 Electronic Nose was provided by Airsense in Schwerin, Germany. A TS-5000Z Electronic Tongue System was purchased from Insent in Kyushu, Japan. A Thermo1300 Gas Chromatography–Mass Spectrometry (GC-MS) Instrument was purchased from ThermoFisher in Waltham, MA, USA. A BSA224S Electronic Analytical Balance and BS124S Electronic Balance were purchased from Sartorius in Gottingen, Germany.

### 2.3. Methods

#### 2.3.1. Sample Preparation

The fresh large yellow croakers were slaughtered, and the head, scales, and viscera were removed, while the back muscles were cut into fillets with dimensions of 2 cm × 2 cm × 1.5 cm. The water content was measured to be 77% and recorded as MC-77. After marinating with 3% salt for 3 h, the water content was determined to be 70%, recorded as MC-70. The pilot experiment found that when the marinated fish slices were dried in hot air at 45 °C for 1.3, 2.08, and 3.08 h, the moisture content of the fish slices was measured to be 65%, 60%, and 55%, respectively. Then, the fish fillets with different water contents after hot air drying were placed in a preheated oven and roasted at 220 °C for 20 min. The fillets were recorded as BMC-77, BMC-70, BMC-65, BMC-60, and BMC-55, respectively. After roasting and cooling to room temperature, the relevant indicators were measured.

#### 2.3.2. Color and Luster

The lightness value L*, redness value a*, and yellowness value b* of the roasted large yellow croaker fillets with different water contents were determined using the method of Zhang et al. [29].

#### 2.3.3. Hardness and Shear Force

The hardness and shear force were measured according to the methods of Li [27]. An A/MBL probe (TA-XT Plus; Stable Micro System, London, UK) was used, and the pre-test speed was 1 mm/s, the test speed was 1 mm/s, the test speed was 1 mm/s, the test speed was 1 mm/s, the deformation was 50%, the trigger force was 5 g, and each group of samples was measured in parallel 6 times.

#### 2.3.4. TBARS

The method of Cheng [30] was used with slight modifications. The large yellow croaker fillets were ground, and 5 volumes of 20% trichloroacetic acid were added. The mixture was vortex oscillated for 1 min, centrifuged at 8000 rpm 4 °C for 20 min, filtered using filter paper, and the volume was adjusted to 50 mL. A 5 mL volume of the filtrate was mixed with 5 mL of a 0.02 MTBA solution, which was then boiled in a water bath for 40 min. The absorbance was measured at 532 nm. Each group of samples was measured in parallel 4 times.

#### 2.3.5. TVB-N

The TVB-N analysis was performed according to the method of Niu et al. [31] with slight modifications. First, 5 g of the sample was crushed, and 5 mL of distilled water was added. The mixture was mixed well and then allowed to stand for 30 min before the supernatant was filtered using a four-layer filter cloth. A 1 mL volume of the supernatant was mixed with 1 mL of a saturated K_2_CO_3_ solution in the outer chamber of a Conway dish. A 1 mL volume of a boric acid solution and 1 drop of the mixing indicator (methyl red ethanol and bromocresol green ethanol mixture) were added to the inner chamber of a Petri dish. After incubation at 37 °C for 2 h, the resulting mixture in the chamber was titrated with 10 mM HCl until a pale pink color appeared. The TVB-N content (mg/100 g) was calculated using the following formula:

(1) $$\mathrm{TVB}\text{-}N\;(\mathrm{mg}/100\;g)=\frac{\left(V_{1}-V_{2}\right)\times C\times 0.14\times d\times 100}{m}$$

Therein, the following are defined:

V_1_—volume of hydrochloric acid in the test solution;
V_2_—hydrochloric acid volume in reagent blanks;
m—sample weight;
C—hydrochloric acid concentration;
d—dilution factor.

#### 2.3.6. Water Distribution

The water distribution of the roasted large yellow croaker was analyzed using a MesoMR LF-NMR analyzer (MesoMR23-060H-1; Shanghai Niumag Analytical Instrument, Shanghai, China) using the method of Rui [32] with slight modifications. The large yellow croak fillets were put into nuclear magnetic tubes, and T2 sampling was performed using the following CPMG pulse sequence parameters: SF = 21 MHz, O1 = 63,178.75 Hz, P1 = 6 μs, and P2 = 10 μs. The parameters were adjusted as follows: SW = 125 kHz, TW = 2500 ms, RFD = 0.08 ms, RG1 = 20, DRG1 = 2, NS = 8, PRG = 0, NECH = 3300, and TE = 0.15 ms. The transverse relaxation time T2 map was obtained after iterative inversion; each sample was measured three times in parallel.

The large yellow croaker fillets were placed in the center of a 60 mm diameter RF coil, and magnetic resonance images were obtained using the imaging software of the MRI analyzer. The image parameter settings were as follows: TR = 2000 ms and TE = 18.125 ms. False color images were drawn using Osirix software (OsiriXLifev.7.0.4; Geneva, Switzerland).

#### 2.3.7. Electronic Nose Analysis

The aromas of the roasted large yellow croaker were investigated using a PEN3.5 electronic nose (Airsense Analytics GmbH, Schwerin, Germany) using the method of Jiang [33] with modifications. A 5 g sample of the large yellow croaker fillets was chopped, transferred to a headspace bottle, sealed, and left for 2 h for electronic nose detection. The measurement parameters were as follows: sampling time of 1 s, testing time of 180 s, sensor cleaning time of 200 s, and flow rate of 400 mL/min. Each group of samples was tested 4 times. PEN 3.5 winmuster software was used for data acquisition and processing and principal component analysis (PCA); clustering discriminant analysis of the different samples was performed.

#### 2.3.8. Electronic Tongue Analysis

The taste profiles of the roasted large yellow croaker were generated using a TS-5000Z electronic tongue (Insent, Atsugi-shi, Japan) and the method of Wu et al. [34] with modifications. The large yellow croaker fillets were minced and weighed (70.0 ± 0.1 g) into a beaker. A 210 mL volume of deionized water was added before the mixture was stirred for 30 min, centrifuged for 10 min at 10,000 r/min at 4 °C, and filtered; 30 mL of this superserum was used in the analysis. Before analyzing the sample, the sensor was first cleaned for 90 s with a cleaning solution and then with a reference solution (120 + 120 s); it was then immersed in the sample solution. The sample was measured for 30 s, collecting data every 1 s, with the response value at 30 s set as the original data of the electronic tongue (at this time, the sensor became stable). The reference solution was a mixture of potassium chloride and tartaric acid to guarantee the reliability of the data. It was measured in parallel 4 times and used in the PCA of the raw data.

#### 2.3.9. Gas Chromatography–Mass Spectrometry (GC-MS)

The volatile compounds were determined using GC-MS (Shimadzu, Tokyo, Japan) according to method in [35]. A 2.00 g sample of the crushed fish was mixed with a saturated sodium chloride solution and transferred to a 10 mL headspace bottle. After heating at 55 °C for 30 min, the activated solid phase microextraction head was inserted and allowed to adsorb for 30 min and desorb for 5 min at the injection port before the GC-MS analysis was performed.

The chromatographic conditions were as follows: a SH-Wax quartz capillary column (30 m × 0.25 mm, 0.25 μm, Shimadzu, Tokyo, Japan); the initial temperature of the column was held at 50 °C for 2 min, the temperature was increased at a rate of 5 °C/min to 180 °C, which was held for 5 min, and then the temperature was increase at a rate of 10 °C/min to 250 °C; the flow rate of the carrier gas (He) was maintained at 1.0 mL/min for 5 min; no shunt injection and an inlet temperature of 250 °C.

The mass spectrum conditions were as follows: electron energy of 70 eV; ion source temperature of 230 °C; quadrupole temperature of 150 °C; transmission line temperature of 280 °C; ion acquisition mode was full scanning mode; and mass range of 35~350 *m*/*z*.

The qualitative analysis was performed as follows: The GC-MS analysis was performed using the standard TMP (2,4,6-collidine; Sigma (Marlborough, MA, USA)), and the relative concentration of each compound was calculated according to Formula (2) and then compared with the NIST database for identification.

(2) $$Relative\;content\;of\;volatile\;flavor\;substance=\frac{Ai\times V_{TMP}{\times C}_{TMP}}{m\times A_{TMP}}$$

Therein, the following are defined:

Ai—peak area of volatile flavor substance;
C_TMP_—concentration of the internal standard;
V_TMP_—volume of the internal standard;
A_TMP_—peak area of the internal standard;
M—quality of the fish sample.

#### 2.3.10. Preparation and Determination of Myofibrillar Proteins

The method of Zhang [29] was used with slight modifications. The roasted fish was crushed, and 4 volumes of 10 mmol/L Tris-HCI (pH 7.2) were added. After 20 s of homogenization, the mixture was centrifuged for 20 min at 4500 r/min. To the precipitate, 4 volumes of a 10 mmoL Tris-HCl buffer (containing 0.6 mol/L NaCl, pH 7.2) were added; the mixture was mixed well, homogenized for 90 s, and centrifuged at 4500 r/min for 20 min. The supernatant was a solution of myofibrillar proteins.

The concentration of myofibrillar proteins was determined using a BCA kit. The extracted myofibrillar protein solution was diluted to 1 mg/mL, the sample buffer solution was added at a volume ratio of 1:4, and the mixture was mixed well. The supernatant was boiled in a water bath for 5 min, centrifuged at 10,000 r/min for 1 min, and then used as a sample for electrophoresis. A 15.0 uL volume of a 12% separation glue and 5% concentration glue mixture were added to the sample in each lane. The samples were subjected to 200 V constant pressure electrophoresis until the indicator reached the bottom of the rubber plate. The samples were incubated in the Coomax R250 staining solution for 30 min, and after decolorization, photos were taken using a scanner and analyzed.

#### 2.3.11. Sensory Evaluation

Using the sensory requirements of Chen et al. [36] with slight modifications, the taste and odor of the roasted large yellow croaker fillets were evaluated and scored on a scale of up to 9 points according to Table 1. Ten food students, between 20 and 30 years old, were invited to form the sensory evaluation team. All samples were shuffled and renumbered prior to the evaluation to avoid possible confounding effects.

### 2.4. Statistical Analysis

All experiments results are expressed as the mean ± SD. SPSS Statistics 26.0 (Chicago, IL, USA) was used for the statistical analyses. The significance of the main effects (*p* < 0.05) was determined using Duncan’s multiple comparison test. The principal component analysis and Pearson correlation analysis of the quality characteristics were carried out using Origin 2023 (OriginLab Corp., Northampton, MA, USA). The cluster analysis of the volatile substances was carried out using Tbtools. The OPLS-DA model was developed and analyzed using Simca14.1.

## 3. Results and Discussion

### 3.1. Changes in Color of Large Yellow Croaker Fillets with Different Water Contents After Roasting

Color is an important indicator of product quality, and changes in the color of fish fillets during processing may be related to water evaporation, browning chemical reactions, caramelization, and the formation of a hard skin shell [37]. Table 2 shows the color of the roasted large yellow croaker fillets with different water contents. Changes in the L*, a*, and b* values were used as the main indicators of the color difference and lightness of the fish. The larger the a* value is, the redder the color; the larger the b* value is, the yellower the color [38]. As the water content decreased, the L* value of the fish fillets after roasting decreased significantly (*p* < 0.05), from 69.43 to 63.24, resulting in a decrease of 8.92%. This may have been due to two reasons: It could have been due to the Maillard reaction, which produces brown pigment and darkens the fish color, or it may be that the water content of the fish was reduced during processing, which reduced the reflection of light. The whiteness value also decreased with decreasing water content. However, the a* and b* values showed significant upward trends with decreasing water content (*p* < 0.05). The b* value of BMC-55 reached 22.59, which was 1.87 times that of BMC-77. This may be due to the increase in the a* value caused by the oxidation of ferrimyoglobin in the fish fillets during processing, while the continuous increase in lipid oxidation in the fish fillets led to a significant increase in the b* value. Similarly, Li et al. [39] found that as the water content decreased, the L* value of roasted shrimp decreased, while the a* and b* values increased. Based on the sensory evaluation and the color of the fillets, BMC-60 and 65 showed no significant differences (*p* > 0.05), but BMC-60 formed a golden and crispy shell after roasting, and the color was more attractive.

### 3.2. Changes in Texture of Large Yellow Croaker Fillets with Different Water Content After Roasting

**Figure 1:**
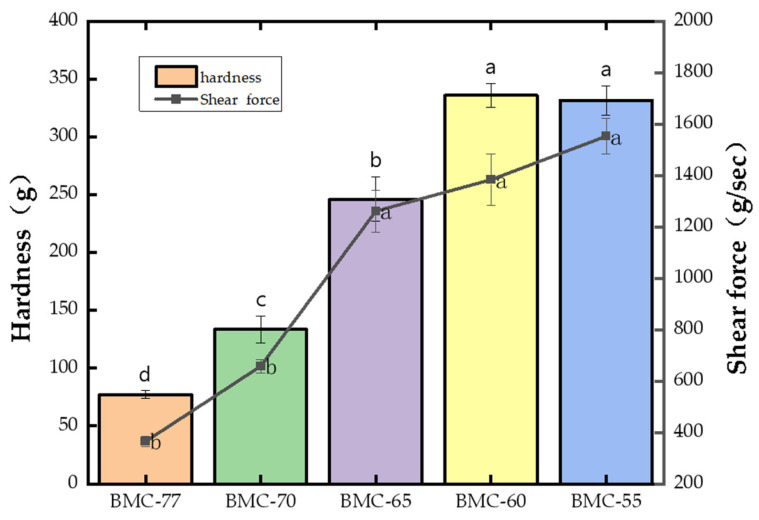
Effect of different water content on texture of large yellow croaker fillet. (a,b,c,d: indicate that different treatments have significant differences, *p* < 0.05).

Hardness and shear force are important performance indicators of the texture of fish fillets. It can be seen from Figure 1 that the hardness and shear force of the large yellow croaker fillets significantly increased as the fish water content decreased (*p* < 0.05). When the water content decreased to 65% (BMC-65), the hardness and shear force were 246.24 and 1260.92, respectively, which were 2.19 times and 2.46 times higher than that of BMC-77. These values were also significantly higher than those of BMC-70 (*p* < 0.05). However, at lower moisture contents, the changes in shear force and hardness were no longer significant (*p* > 0.05). The shear forces of BMC-60 and BMC-55 reached 1383.23 and 1552.86 g/sec, respectively, and their hardness increased by 258.75 and 254.29 g compared to BMC-77, indicating that after salting and hot air drying, the water content of the fillets could be reduced, and the texture of the fillets could be significantly changed. This is because after fish is pickled and dried using hot air, the myofibrillar fibers become shorter, and the length of the sarcomere becomes shorter, which makes the fish dry and more tough [40]. At the same time, the evaporation rate of water on the surface of the fish fillet is greater than that of water spreading from the inside to the skin. The skin of the slices loses water and shrinks rapidly to form a dense and compact hard shell. As the baking continues, the formation of the hard skin shell increases, resulting in changes in the texture of the fish and an increase in the shear force. This is consistent with the results of Serra et al. [41], who found that the hardness and chewable shear force indexes of meat products were negatively correlated with their water content.

### 3.3. Changes in TBARS Content of Large Yellow Croaker Fillets with Different Water Contents After Roasting

**Figure 2:**
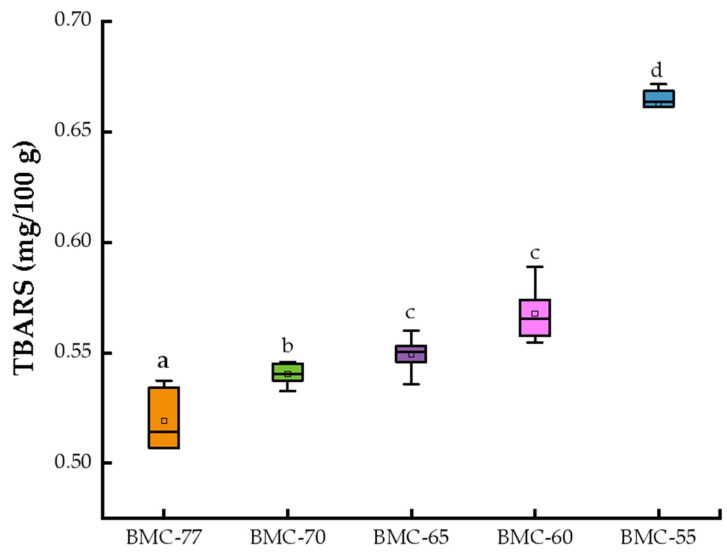
Effect of different water content on TBARS of large yellow croaker fillet. (a,b,c,d: indicate that different treatments have significant differences, *p* < 0.05).

The degree of fat oxidation is closely related to the sensory quality and flavor quality of food products [42]. During the processing of the product, heat and O_2_ will accelerate the fatty hydrolysis and oxidation reactions of fish, resulting in lipid peroxidation and the production of malondialdehyde and other lipid peroxidation products. The resulting malondialdehyde can combine with thiobarbituric acid to form a stable complex at high temperatures; this complex’s maximum absorption is 532 nm [43]. Therefore, the TBARS content of the large yellow croaker fillets with different water contents after roasting was determined, and the results are shown in Figure 2. The lower the water content of the fillets, the higher the TBARS content, which ranged from 0.52 mg/100 g to 0.66 mg/100 g. The TBARS content of BMC-65 was 1.27 times higher than that of the BMC-55 fish, indicating that the salting and hot air drying had a certain effect on the oxidation of fish fat. There was no significant difference between the TBARS contents of BMC-65 and BMC-60 (*p* > 0.05), but these values were significantly lower than that of BMC-55 (*p* < 0.05). As the water content decreased, the TBARS content of the fish meat increased, which means that fat oxidation increased, and unpleasant odors were produced. This will seriously affect the flavor of the fish, since fish lipids exposed to the air for a long period of time will react with oxygen in the air to form peroxides, which are further decomposed to produce aldehydes and other substances [44]. This experimental result is similar to that of Xu et al. [45], who showed that a heat pump cold air drying process at 8~35 °C had a great effect on the fat oxidation of silver carp after being exposed to the air for a long period of time.

### 3.4. Changes in TVB-N Content of Large Yellow Croaker Fillets with Different Water Contents After Roasting

As can be seen from Figure 1, after the salting and drying treatment, the TVB-N content of the fish increased significantly as the water content decreased (*p* < 0.05), and the values were all less than 30 mg/100 g. The TVB-N content of the roasted fish was higher than that of the treated fish. A possible reason for this is that the proteins in the fish was further decomposed into amines by the Maillard reaction and lipid oxidation during the roasting process.

**Figure 3:**
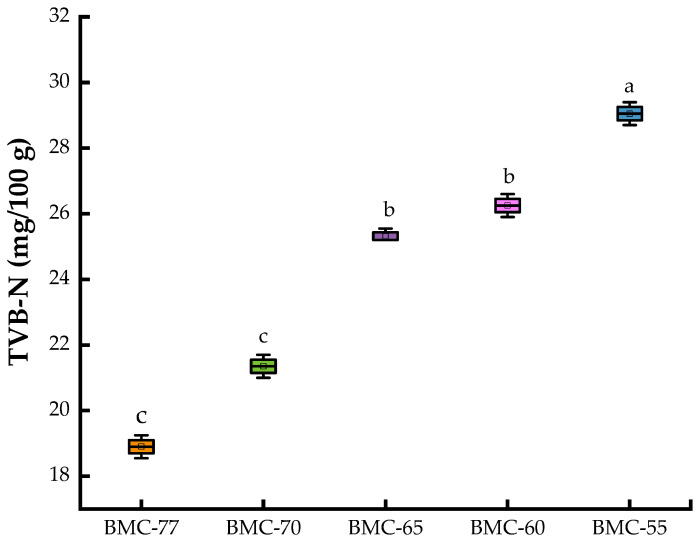
Effects of different water contents on TVB-N value of large yellow croaker fillets. (a,b,c: indicate that different treatments have significant differences, *p* < 0.05).

During the processing of large yellow croaker fillets, due to the growth and reproduction of bacteria and the oxidation and decomposition by enzymes, the proteins are decomposed, and ammonia and amines and other alkaline nitrogenous substances are produced, resulting in fish spoilage. Studies have shown that the sensory acceptance limit in fish is a TVB-N content of 30 mg/100 g [46]. As can be seen from Figure 3, the water content of the large yellow croaker fillets gradually decreased after salting and drying, and the TVB-N content of the roasted fillets significantly increased (*p* < 0.05) from 18.90 to 29.05 mg/100 g due to the further decomposition of the proteins in the fillets into amines under the high temperature. When the water content decreased to 65%, the TVB-N content was similar to that of BMC-60, reaching 26.25 and 25.32 mg/100 g, respectively, and the TVB-N content of BMC-55 was much higher, close to 30 mg/100 g. The reason for this might be that, with the decrease in water content, the proteins in the fish meat were further degraded into alkaline nitrogen-containing compounds by the action of microorganisms, resulting an increased TVB-N content. These degradation products play an active role in the formation of flavor and can produce unique flavors. Our results are similar to those of Chen et al. [47] who used a low-salt wet method to marinate golden pomfret and found that the TVB-N content of the fish meat increased during the curing process, and the proteins were degraded into small-molecule alkaline nitrogen-containing compounds, which affected the flavor.

### 3.5. Influence of Different Water Content on Water Migration Pattern in Large Yellow Croaker Fillet After Roasting

**Figure 4:**
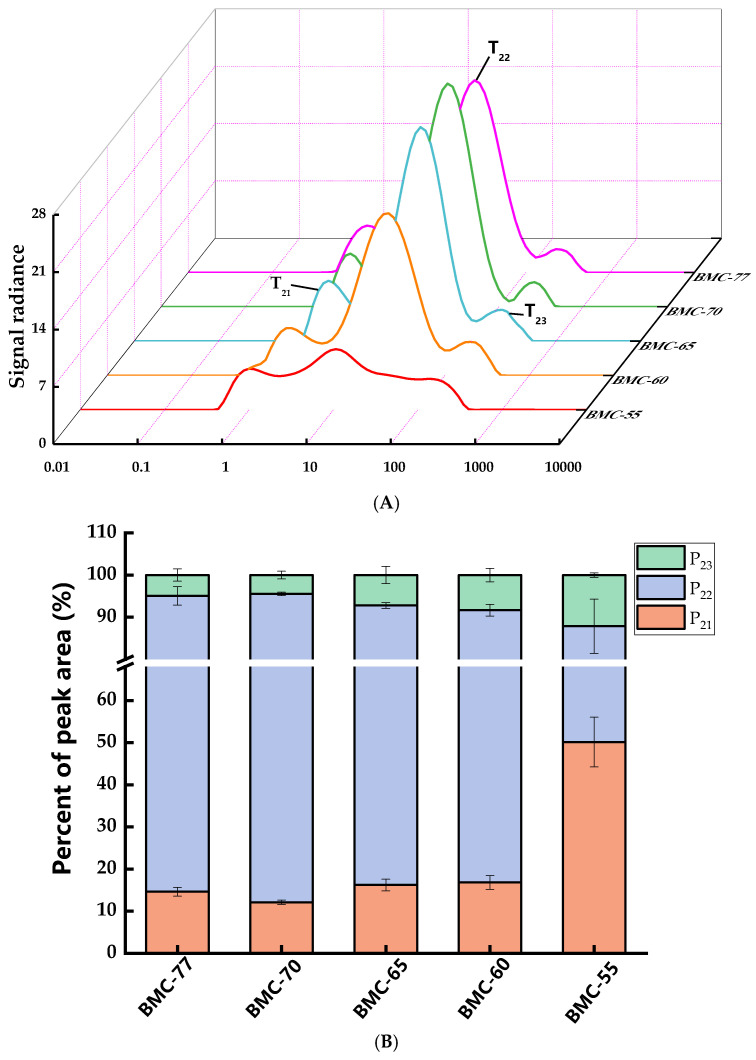

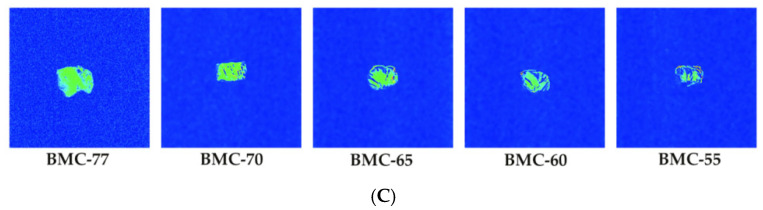
Large yellow croaker fillets water contents (**A**), their peak area proportions (**B**), and pseudo-color images (**C**).

Low-Field Nuclear Magnetic Resonance (LF-NMR) technology can effectively elucidate the state, distribution, and migration characteristics of water within large yellow croaker fillets. The main parameters measured included the transverse relaxation time (T_2_) and signal amplitude. The T_2_ relaxation time can reflect the degree of binding between water and substrates. The smaller the T_2_ value, the closer the binding, the lower the water freedom, and the position of the peak in the T_2_ map will shift toward the left side. The states can be categorized into three types: T_21_ (0–10 ms) for bound water; T_22_ (10–100 ms) for immobilized water within the myofibrillar structure; and T_23_ (100–1000 ms) for free water within the myofibrillar structure [48]. Figure 4A shows the T_2_ inversion spectra of the large yellow croaker fillets with different water contents roasted at 220 °C for 20 min. Compared to BMC-77, the T_21_, T_22_, and T_23_ of BMC-65 were significantly decreased (*p* < 0.05). The T_22_ of BMC-60 and BMC-55 were significantly lower compared to that of the BMC-65 fillets (*p* < 0.05). This is consistent with the trends in the T_2_ inverse spectrum, in which the BMC-70 fillets exhibited the highest P_22_ and the lowest P_21_ and P_23_ (Figure 4B). Compared to BMC-70, the P_22_ of BMC-65 decreased by 45.72%, while the P_21_ and P_23_ increased by 38.04% and 7.68%, respectively. This indicates that as the water content decreased, the immobilized water in the fillets tended to transform into free water after roasting. A possible explanation for this is that the myofibrillar contraction during processing prevents the loss of immobilized water and transforms it into free water. Simultaneously, the immobilized water content decreased during roasting, leading to a reduction in its percentage and a significant increase in the percentage of bound water. These findings are consistent with the results of Liu et al. [49] who observed a similar transformation of immobilized water into free and bound water during the roasting of duck. Additionally, Sultana et al. [50] found that the lower the water content of fish fillets, the higher the accumulation of macromolecular reaction products, causing myofibrillar contraction and enhancing the water-binding capacity of the tissue structure. Macromolecular substances such as lipid molecules lack the protection of water molecules, and the area exposed to air is large, making it easy for them to contact oxygen and thereby accelerating their oxidation [51]. These results are consistent with the TBARS results: BMC-55, with its lower water content, had a significantly higher TBARS content than that of BMC-65 (*p* < 0.05). The change in P_21_, P_22_, and P_23_ will also affect the Maillard reaction: if the free water content is too high, the high dilution will slow down the rate of the Maillard reaction, but if the content is too low, the temperature of the reaction system will rise, and the Maillard reaction becomes accelerated. However, the high temperature may lead to excessive reactions, resulting in undesirable flavors and colors. At the same time, the presence state of water is closely related to volatile flavor compounds. Zhang et al. [52] found that changes in the water state in food affect the protein structure and change the binding sites of proteins and volatile substances, thereby affecting the production of volatile flavor compounds. Some studies have found that the immobile water content is positively correlated with the isovaleraldehyde and heptanaldehyde contents and negatively correlated with the nonanal content [31].

MRI was used to obtain two-dimensional images of the internal morphological organization of the fish fillets. In Figure 4C, the color shading indicates the signal strength and brighter colors indicate more protons. It can be observed that the lower the water content of the fish fillet, the smaller and more uneven the water distribution after roasting was, with more moisture in the center and less moisture in the periphery. This may be due to the damage to the protein tissue of the fish fillet before salting and drying, which makes the water in the fillet combine with macromolecular compounds; the outer part is heated first, and then the heat is transmitted to the inside during the roasting process, resulting in greater loss of external water and less internal water loss. This result is consistent with that of Zhang et al. [53], who found that the moisture of shrimp meat was unevenly distributed during thermal processing, with higher internal moisture and lower peripheral moisture.

### 3.6. Electronic Nose Analysis Results for Large Yellow Croaker Fillets with Different Water Contents After Roasting

**Figure 5:**
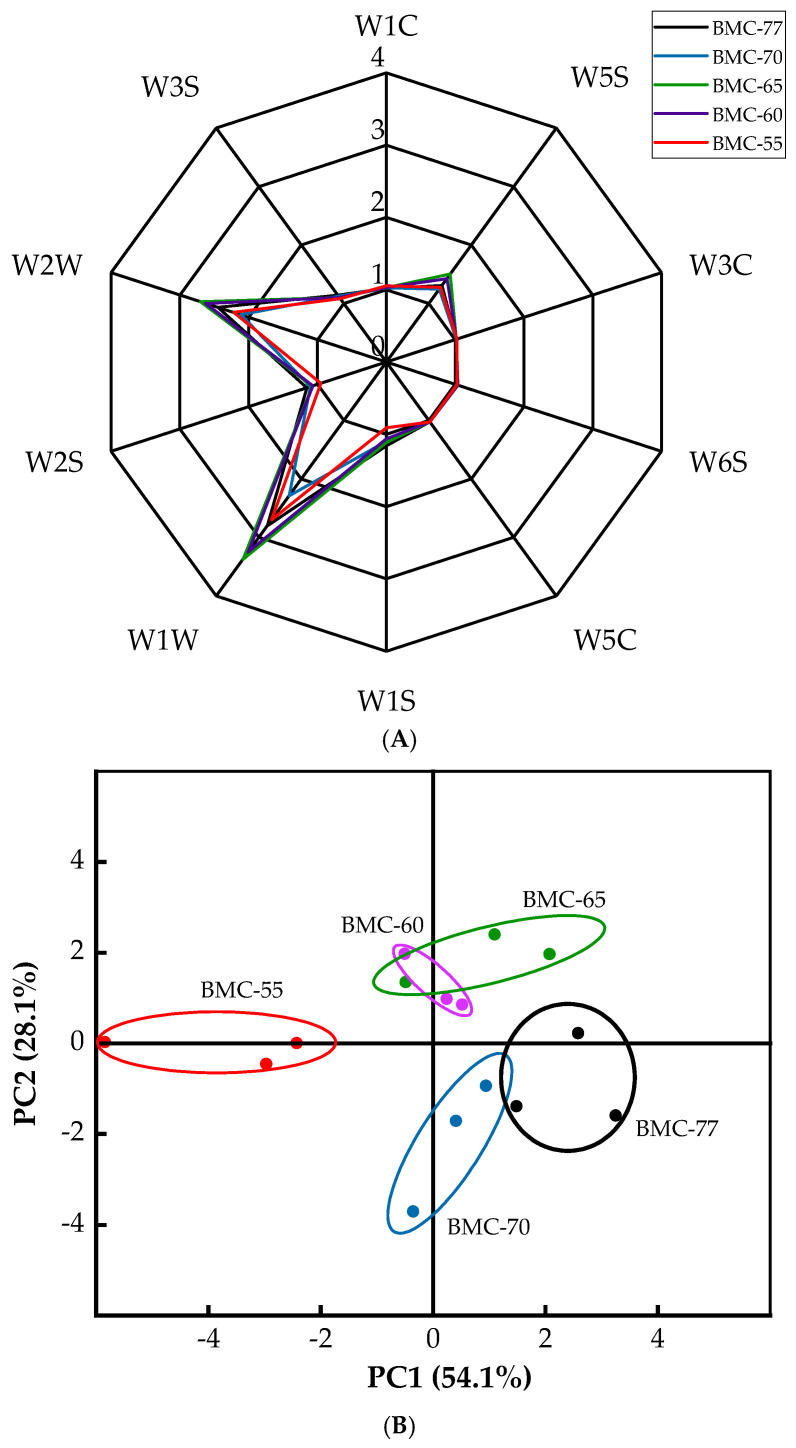
Radar diagram (**A**) and PCA diagram (**B**) of large yellow croaker roasted fish fillet.

Electronic noses are a common tool for identifying and analyzing the aroma of samples. PCA is a mathematical dimensionality reduction technique for visualizing the differences between groups of samples [54]. The radar charts of the odor profiles generated using 10 odor sensors for the large yellow croaker fillets with different water contents are shown in Figure 5A. The results showed that the W1W (sensitive to sulfides), W2W (sensitive to aromatic components and organic sulfides), and W5S (highly sensitive to nitrogen oxides) sensors exhibited the strongest response to volatile compounds in the roasted large yellow croaker fillets. Notably, in the BMC-65 samples, the W5S, W6S, W1W, and W2W odor values were significantly higher than those of the other groups, reaching 1.51, 1.04, 3.37, and 2.70, respectively.

The PCA results for the responses of the electronic nose sensor for the large yellow croaker fillets with different water contents are shown in Figure 5B. The contribution rates of the first principal component (PC1) and the second principal component (PC2) were 54.1% and 28.1%, respectively, with a cumulative variance contribution rate of 82.2%, indicating that these two principal components could effectively represent the overall aroma characteristics of each group of large yellow croaker fillets. The distribution areas of the odor values of the fresh fillets and marinated samples overlapped, suggesting that there were no significant differences in their aromas. However, both groups showed distinct distribution regions compared to the other samples, indicating that there was a significant difference in aroma profile between the samples that underwent the drying treatment and those that did not.

### 3.7. The Effect of Different Water Content on Electronic Tongue Analysis of Large Yellow Croaker Fillet After Roasting

**Figure 6:**
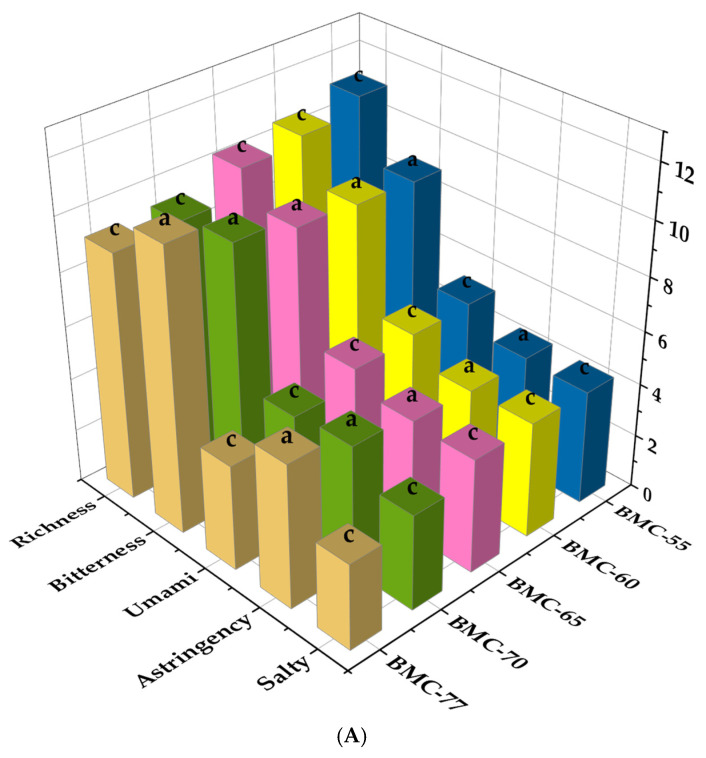

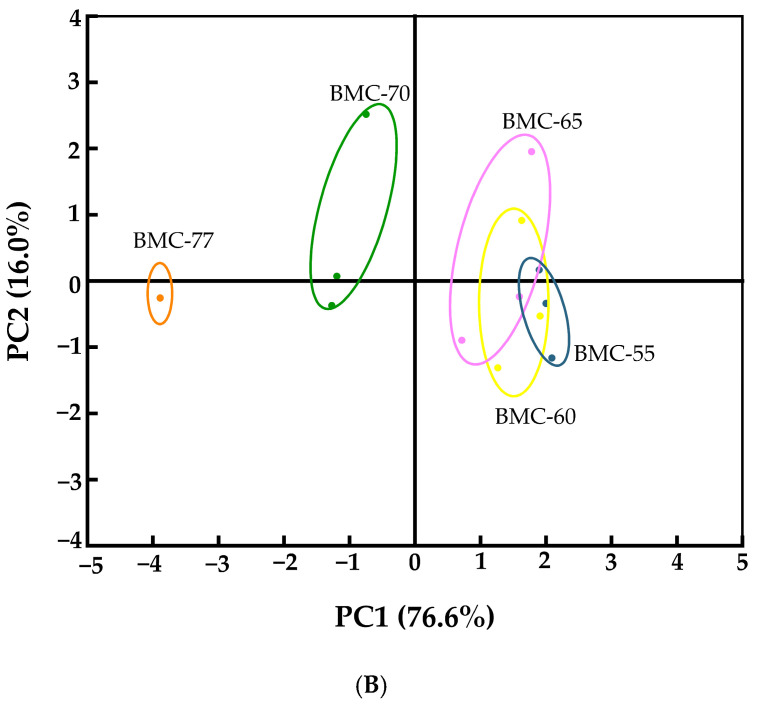
Histogram (**A**) and PCA diagram (**B**) of large yellow croaker roasted fish fillet. (a,b,c: indicate that different treatments have significant differences, *p* < 0.05).

Electronic tongues are intelligent bionic taste systems that simulate the taste buds on the human tongue, converting electric potential values into taste values. They can distinguish and quantify basic tastes such as sourness, sweetness, bitterness, umami, and saltiness in fish fillets. Therefore, an electronic tongue was used to measure the taste differences in the roasted large yellow croaker fillets with different water contents, and the taste profiles of the fillets were constructed using the response intensities of the sensors. As can be seen from Figure 6A, the water content of the fillets significantly influenced the bitterness, astringency, astringent aftertaste, richness, saltiness, and umami after roasting (*p* < 0.05). Significant differences were observed in umami, richness, and saltiness between the BMC-77 and BMC-65 groups (*p* < 0.05), but no significant differences were found between the BMC-65, BMC-60, and MC-55 groups (*p* > 0.05). When the water content of the fillets was 65% (BMC-65 group), the umami value reached 5.12 after roasting, which was 1.29 times that of the BMC-77 group, and the richness increased by 11.45 compared to the BMC-77 group. Similarly, the saltiness of the BMC-65 group was 1.33 times that of the BMC-77 group, while the astringency value was significantly reduced by 18.15% compared to the BMC-77 group. These results indicate that the salting and drying process altered the water content of the fillets, thereby affecting their taste components after roasting. The electronic tongue results were analyzed using PCA. Figure 6B shows that PC1 (76.6%) and PC2 (16.0%) accounted for 92.6% of the total variance, indicating that the model can explain the results of all the samples. As the water content decreased, the projection (VIP) of the samples shifted toward the positive axis of PC1, suggesting that the salting and drying processes significantly altered the taste characteristics of the large yellow croaker fillets. The projections of the BMC-65 group overlapped with those of the BMC-60 and BMC-55 groups, indicating that these groups had similar taste characteristics. The results demonstrated that the taste of the roasted yellow croaker fillets changed due to the differences in water content, with significant differences between the BMC-77 and BMC-70 groups compared to the BMC-65 group (*p* < 0.05) but no significant differences between the BMC-65, BMC-60, and BMC-55 groups (*p* > 0.05).

### 3.8. The Effect of Different Water Contents on Volatile Components of Large Yellow Croaker Fillets After Roasting

**Figure 7:**
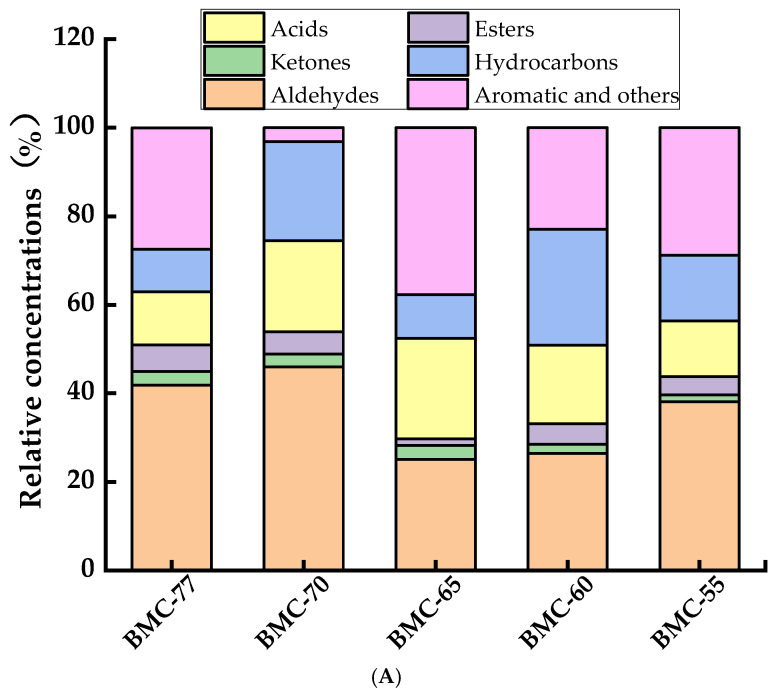

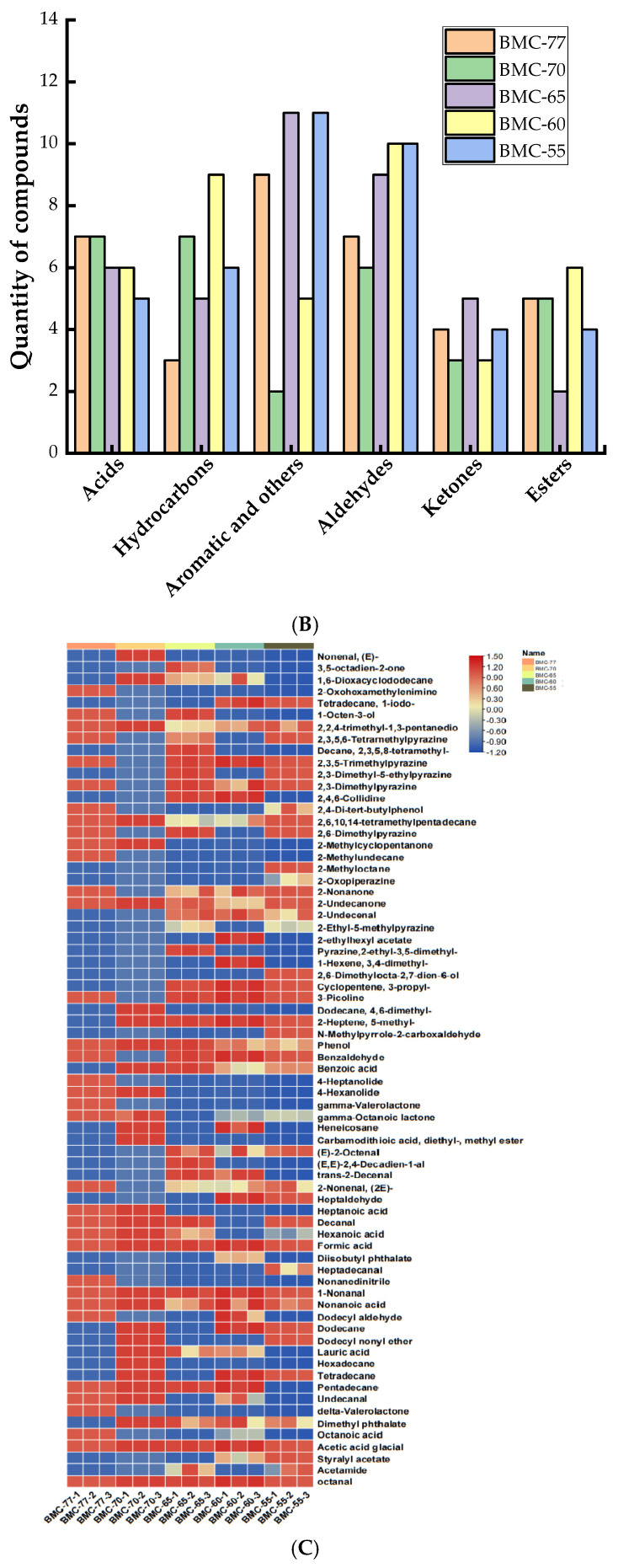
The relative concentrations (**A**), number types (**B**), and heat map (**C**) of volatile flavor compounds with different water contents.

Under normal circumstances, high temperatures will induce lipid oxidation in fish fillets, generating a variety of aldehydes and ketones. Aldehydes are relatively abundant and have low volatilization thresholds, allowing them to significantly contribute to the aroma of fillets. Therefore, the volatile components in the roasted fillets with different water contents were analyzed using GC-MS, which identified a total of 70 volatile components, mainly aldehydes, alcohols, acids, hydrocarbons, esters, aromatics, and other compounds. BMC-77, BMC-70, BMC-65, BMC-60, and BMC-55 were found to contain 35, 30, 38, 39, and 40 volatile compounds, respectively. As shown in Figure 7A,C, compared to the other volatile substances, the relative aldehyde content in BMC-65 reached 25.03%, which was significantly lower than that of the other groups (*p* < 0.05), while the relative content of aromatic and other compounds was the highest in this group at 37.74%. These compounds included 2-ethyl-5-methylpyrazine, 2-ethylalkyl-3, 5-dimethylpyrazine, 2,6-dimethylpyrazine, 2,4,6-trimethylpyridine, and 2, 3-dimethyl-5-ethylpyrazine. Aromatic substances, such as pyridine and pyrazine, are derivatives of the Maillard reaction [55] and impart nutty and toasty aromas [56,57]. The aldehydes in fish fillets are influenced by salting, drying, and roasting processes, which can accelerate the oxidative degradation of fatty acids [58], while some aldehydes can be oxidized and degraded into alcohols, heterocycles, or other volatile compounds.

The GC-MS results are consistent with the TBARS results: as the water content decreased, the TBARS content increased, indicating that a higher degree of lipid oxidation and more volatile flavor compounds (e.g., irritating aldehydes such as propionaldehyde, hexanal, etc.), which may produce undesirable odors. At the same time, some aromatic compounds, such as pyrazines and pyridines, may be generated through the Maillard reaction and lipid oxidation [59,60], which promote the formation and retention of flavor compounds.

As can be seen from Figure 7B, 7, 6, 9, 10, and 10 aldehydes were detected in the five groups, including heptyl aldehyde, capric aldehyde, benzaldehyde, and nonal. The BMC-70 group, which underwent salting before roasting, exhibited accelerated lipid oxidation, leading to an increase in the production of certain aldehydes (Figure 7A), but the variety of aldehydes was significantly lower than that in BMC-77. After salting and drying, the reduction in the water content resulted in an increase in the relative concentration of aldehydes since the hot air drying and roasting promoted the thermal oxidative decomposition of lipids, producing more aldehydes and increasing their variety. Gao et al. [61] also found that the variety of aldehyde compounds in air-dried hairtail gradually increased.

As shown in the heat map in Figure 7C, the relative concentration of nonylaldehyde in the BMC-65 group decreased by 205.6 ng/10 g compared to the BMC-77 group, while the contents of flavor aldehydes, such as heptal aldehyde and trans-2-octenal, increased in the treated groups compared to BMC-77. The relative ketone content in the five groups ranged from 1.55% to 3.21%; the detected ketones included (E,E)-3, 5-octanediene-2-one,1, 6-dioxacyclododecyl-7, 12-dione, 2-methylcyclopentanone, 2-piperazinone, 2-nonanone, 2-undecanone, 5-dodecyldihydro2 (3H) -furanone, alpha-pyranone, and tetrahydro2h-2-pyranone. BMC-65 contained the highest variety of ketones. Ketones possess distinct aromas, producing fatty and burnt notes; they are produced through lipid oxidation and play a significant role in the formation of the roasted aroma of fish fillets [62].

**Figure 8:**
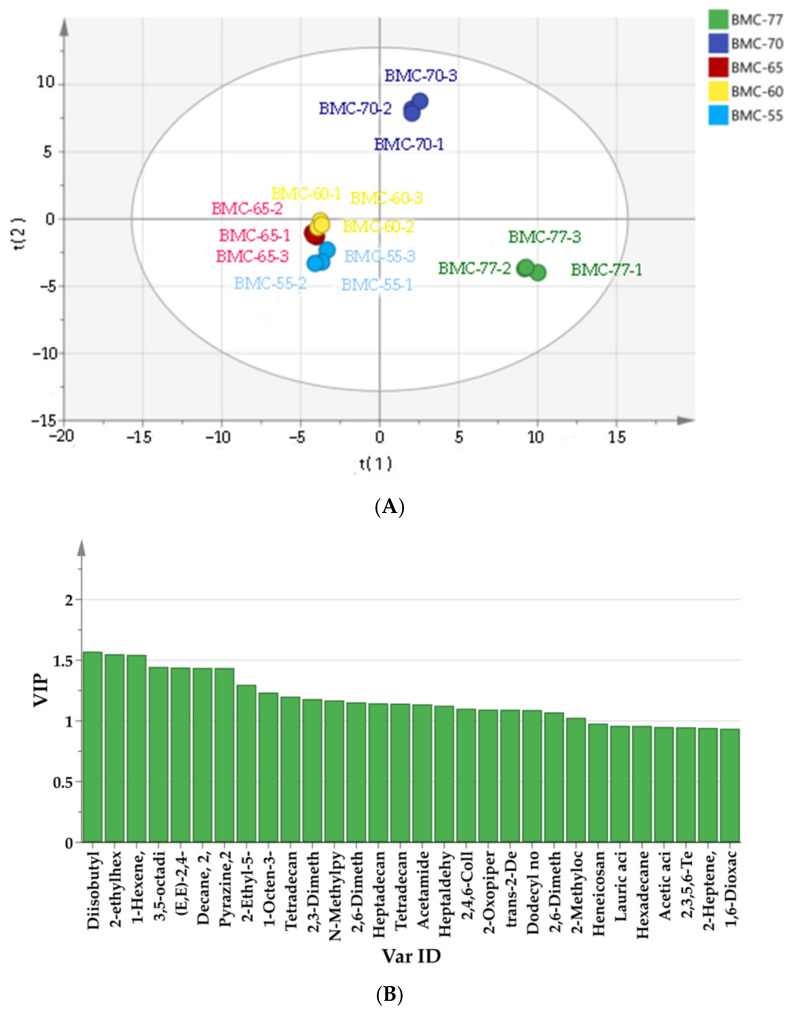
Score (**A**) and VIP (**B**) plots of volatile components of roasted fish fillets with different water contents.

To further determine how the different volatile organic compound profiles led to the differences between the samples, a partial least squares discriminant analysis (PLS-DA) model was generated [63]. The goodness-of-fit parameters (R2X), model explanatory ability (R2Y), and predictive ability (Q2) were 0.942, 0.994, and 0.986, respectively, indicating that the model had good robustness and predictive ability. According to Figure 8A, the five groups could be classified into three categories: BMC-77 was concentrated in the fourth quadrant; BMC-70 was concentrated in the first quadrant; and BMC-65, BMC-60, and BMC-55, which showed no significant differences, were concentrated in the third quadrant. Then, the parameters of the five groups were further distinguished using the Variable Importance in Projection (VIP) method. The higher the VIP value of a variable, the more significant its contribution is in distinguishing between the groups. As shown in Figure 8B, a total of 23 compounds with a VIP value >1 were identified, including heptyl aldehyde, decylaldehyde, diisobutyl phthalate, acetamide, dodecyl aldehyde, dodecane, heptaaldehyde, trans-2,4-decenal, trans-2-decenal, n-methyl-2-pyrrolidine, 2-ethyl-5-methylpyrazine, 2-ethylhexyl acetate, 2-ethyl-3-dimethyl-1-hexene, 3,4-dimethyl-1-hexene, 3,7-dimethyl-1,6-octanediene-3-ol,2-piperazinone, 2,6-dimethylpyrazine, 2,4,6-trimethylpyridine, 2,3-dimethyl-5-ethyl pyrazine, 2,3,5,8-tetramethyldecane, 1-octane-3-ol, (E,E)-3,5-octanediene-2-one, and dodecyl nonyl ether. These compounds can serve as markers for the aroma compounds in roasted large yellow croaker fillets [64].

### 3.9. Effect of Water Content on Myofibrillar Proteins in Large Yellow Croaker Fillets After Roasting

**Figure 9:**
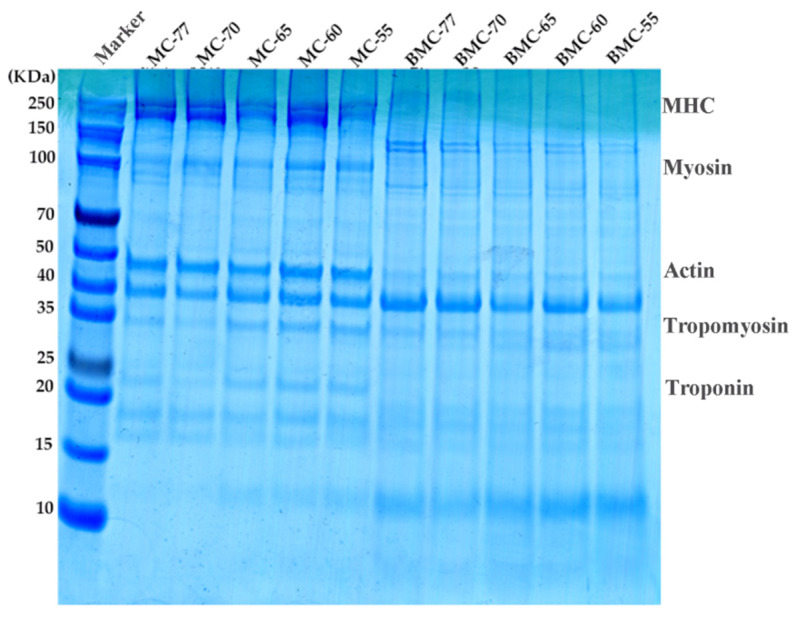
Effect of different water content amounts on protein composition of large yellow croaker roasted fish fillet.

Myofibrillar proteins (MPs) are the most important class of proteins in fillets, accounting for 55% to 60% of the total protein content. In fish fillets, the main MPs are myosin heavy chain (MHC; 200 kDa), actin (48 kDa), troponin I (23 kDa), and tropomyosin (35 kDa). In order to investigate the effect of the water content on the denaturation and degradation of proteins in roasted large yellow croaker fillets, the relative molecular weights of MPs and the number of subunits in the protein were determined using SDS-PAGE. As can be seen from Figure 9, the protein bands of the large yellow croaker fillet samples showed little difference after undergoing the pre-treatments (salting and drying) that caused varying degrees of dehydration. However, after roasting, degradation of MHC, myosin, and actin was observed, indicating that high temperatures reduce the thermal stability of myosin and actin, accelerating protein degradation.

The MHC bands were clearly visible, while the actin bands remained largely unchanged. The tropomyosin bands and troponin I bands in BMC-65, BMC-60, and BMC-55 gradually increased in intensity as the drying degree increased, indicating that a heating treatment could cause the degradation of macromolecular proteins. These findings are consistent with the results reported by Zhang et al. [65].

### 3.10. Effect of Water Content on Sensory Evaluation of Roasted Large Yellow Croaker Fillets

Sensory evaluations provide an intuitive analysis and description of the texture, color, and appearance of fish fillets, reflecting the acceptance and preference of consumers [62]. Therefore, sensory evaluations were conducted on the large yellow croaker fillets with different water contents after roasting at 220 °C for 20 min, and the results are shown in Table 3. As the water content decreased, the individual scores for appearance, aroma, color, and overall sensory evaluation initially increased and then decreased. The BMC-60 group received the highest score of 6.75, while the BMC-65 and BMC-60 groups showed no significant difference in total sensory score (*p* > 0.05). The BMC-77 group had the lowest sensory scores, which were 40.15% and 37.94% lower compared to the BMC-60 and BMC-65 groups, respectively. According to the results from the electronic nose, electronic tongue, TBARS, and TVB-N analyses, the roasted large yellow croaker fillets with a water content of 65% exhibited the best quality in terms of texture, color, aroma, and appearance.

### 3.11. Correlation Analysis of Quality Indicators in Roasted Large Yellow Croaker Fillets with Different Water Content

**Figure 10:**
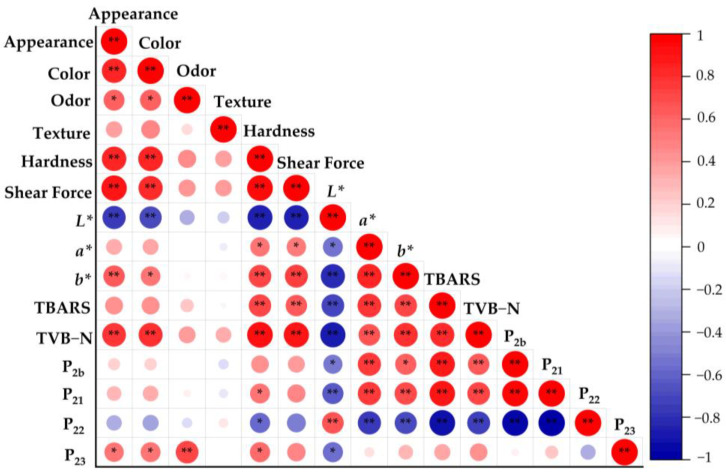
Correlation analysis of quality characteristics of roasted fish fillets of large yellow croaker with different water contents (*: *p* > 0.05; **: *p* < 0.01).

To explore the relationships between the various quality indicators of large yellow croaker fillets, a Pearson correlation analysis was conducted on the sensory evaluation (appearance, color, aroma, and taste), texture (hardness, shear force), color change (L*, a*, and b* values), water distribution (P_21_, P_22_, and P_23_), TBARS, and TVB-N data. The results are shown in Figure 10. The color changes (L*, a*, b*) exhibited a high correlation with lipid oxidation and fillet freshness: L* showed significant negative correlations with the TBARS content, TVB-N content, and P_21_ (*p* < 0.05, *p* < 0.01), while a* and b* showed significant positive correlations with the TBARS content, TVB-N content, and P_21_ (*p* < 0.05, *p* < 0.01). As the water content decreased, the volume of the fillet experienced shrinkage and water loss. Simultaneously, proteins underwent continuous degradation, generating more peptides and amino acids, which could participate in the Maillard reaction. As protein and lipid oxidation increased, the color of the large yellow croaker fillets darkened and turned yellowish. Similar results were observed by Gao et al. [66] in their study on the relationship between protein and quality changes after hot-air drying Pacific white shrimp. Additionally, the appearance of the fillets also showed a significant positive correlation with the hardness and shear force (*p* < 0.05, *p* < 0.01). As the water content decreased and the drying increased, a hard shell formed on the surface of fillets, leading to increased texture and shear force, which was consistent with the results of Rodrigues et al. [67].

## 4. Conclusions

This study investigated the effects of water content on the quality characteristics of roasted large yellow croaker fillets. The fish fillets with a higher initial water content exhibited lower sensory scores after roasting, which were primarily characterized by a soft texture and insufficient flavor. In contrast, fillets with a moderate initial water content, such as BMC-60 and BMC-65, showed the best sensory qualities after roasting, with improved texture, flavor, and overall acceptability. The fillets with a lower initial water content showed a significant increase in b* values (yellowness) and a decrease in a* values (redness) after roasting, indicating that a lower water content contributes to a brighter roasted appearance, which may be related to an enhanced surface Maillard reaction due to the evaporation of water. As the initial water content decreased, the proportion of immobilized water in the fillets significantly decreased (*p* < 0.05), while the proportion of strongly bound water, free water, and loosely bound water significantly increased (*p* < 0.05). Additionally, the hardness and shear force of the roasted fillets significantly increased (*p* < 0.05), suggesting that appropriately reducing the initial water content of fillets can improve the post-roasting texture properties and enhance the mouthfeel. The electronic tongue, electronic nose, and GC-MS results effectively distinguished the five groups of fillets with different water contents. The BMC-65 fillets exhibited more pronounced taste and flavor characteristics compared to BMC-77 and BMC-70, but they showed no significant differences compared to the other low-moisture groups. The PLS-DA of the volatile compounds in the five groups revealed significant differences.

In summary, the initial water content significantly influences the quality characteristics of roasted large yellow croaker fillets. Appropriately reducing the initial water content (e.g., to 65%) can improve the texture, sensory qualities, and appearance of the roasted fillets while delaying the degradation of proteins and lipids. This study provides a theoretical basis for optimizing the roasting process for large yellow croaker fillets. In the future, we will continue to optimize the roasting conditions, such as the roasting temperature and roasting time, in order to determine the most suitable roasting conditions. Further studies should focus on the synergistic effect between baking conditions and moisture content to achieve more accurate quality control.

## Figures and Tables

**Table 1:** Sensory evaluation form for pre-roasted large yellow croaker roasted fish fillet.

Parameter	Grading Criteria
Appearance (10%)	The appearance is loose and incomplete
	The appearance is slightly loose but complete, and the structure is tighter
	The appearance is complete, and the structure is tight
Color (30%)	The fish is white in color, without the yellow color of roasting or black or dark brown in color; charred
	The fish are pale yellow or dark brown in color
	The fish pieces are yellow and shiny
Odor (30%)	The odor of fish is not obvious, and there is an unpleasant fishy odor or other peculiar odor
	The fish has a strong flavor and no irritating odor or other peculiar odors
	The fish has a strong aroma and no other peculiar odor
Texture (30%)	The meat is soft, chewy, less elastic, or hard
	The meat is soft and slightly chewy
	Moderate hardness and chewiness; soft and not dry

**Table 2:** Effect of different thawing methods on the color and pH of large yellow croakers.

	L*	a*	b*
BMC-77	69.43 ± 2.69 ^a^	−2.97 ± 0.38 ^bc^	12.10 ± 1.60 ^c^
BMC-70	69.18 ± 2.80 ^a^	−3.65 ± 0.76 ^c^	9.27 ± 1.98 ^c^
BMC-65	63.24 ± 1.35 ^b^	−2.63 ± 0.42 ^bc^	16.29 ± 1.65 ^b^
BMC-60	64.87 ± 1.21 ^b^	−1.90 ± 1.29 ^b^	17.47 ± 3.38 ^b^
BMC-55	64.87 ± 1.21 ^b^	−0.40 ± 0.93 ^a^	22.59 ± 3.15 ^a^

Note: Different letters after the same column value indicate significant difference (*p* < 0.05).

**Table 3:** Effect of different water content amounts on the sensory scores of large yellow croaker after roasting.

Grading Items	Appearance (10%)	Color (30%)	Odor (30%)	Texture (30%)	Total Score (9 Points)
BMC-77	5.50 ± 1.04 ^c^	3.50 ± 0.54 ^b^	4.60 ± 1.11 ^b^	3.50 ± 0.81 ^b^	4.04 ± 0.46 ^c^
BMC-70	6.00 ± 1.27 ^bc^	4.10 ± 0.66 ^b^	5.20 ± 1.53 ^ab^	6.00 ± 1.25 ^a^	5.18 ± 0.63 ^b^
BMC-65	7.40 ± 1.11 ^ab^	6.80 ± 0.41 ^a^	7.20 ± 1.26 ^a^	5.20 ± 1.57 ^ab^	6.51 ± 0.58 ^a^
BMC-60	8.20 ± 0.75 ^a^	7.80 ± 0.61 ^a^	6.40 ± 1.45 ^ab^	6.00 ± 1.72 ^a^	6.75 ± 0.85 ^a^
BMC-55	7.90 ± 0.92 ^a^	6.70 ± 0.98 ^a^	5.60 ± 2.05 ^ab^	5.00 ± 1.71 ^ab^	5.90 ± 0.80 ^ab^

Note: Different letters after the same column value indicate significant difference (*p* < 0.05).

## Data Availability

The data used to support the findings of this study can be made available by the corresponding author upon request.
